# 24-hour movement behaviors and changes in quality of life over time among community-dwelling older adults: a compositional data analysis

**DOI:** 10.1186/s12966-024-01681-9

**Published:** 2024-11-12

**Authors:** Lotta Palmberg, Kristin Suorsa, Antti Löppönen, Laura Karavirta, Taina Rantanen, Timo Rantalainen

**Affiliations:** 1 Gerontology Research Center and Faculty of Sport and Health Sciences, University of Jyväskylä, Jyväskylä, Finland; 2 Department of Public Health, University of Turku and Turku University Hospital, Turku, Finland; 3 Centre for Population Health Research, University of Turku and Turku University Hospital, Turku, Finland

**Keywords:** Physical activity, Sedentary behavior, Sleep, Wellbeing, Compositional data analysis

## Abstract

**Background:**

Favorable movement behavior patterns, comprising more physical activity, less sedentary behavior, and sufficient sleep, may promote the maintenance of good quality of life (QoL) with advancing age. The aim of the present study was to investigate whether movement behaviors predict future changes in QoL among community-dwelling older adults over a four-year follow-up.

**Methods:**

Participants were 75-, 80- and 85-year-old community-dwelling older adults (*n* = 203) followed up for 4 years. Participants wore thigh- and trunk-mounted accelerometers for 3–7 days at baseline. Proportion of time-use in physical activity, standing and sedentary behavior were assessed based on body posture and movement intensity. Time in bed was determined using an automated algorithm. QoL was assessed during a home interview using the short Older People’s Quality of Life Questionnaire at baseline and follow-up (range 13–65, higher scores indicate higher QoL). Compositional linear regression analysis was used to study whether baseline time-use composition predicts changes in QoL.

**Results:**

Over the 4-year follow-up, QoL scores decreased by 5% on average. Higher physical activity in relation to the other movement behaviors was associated with increase in QoL over time (β_ilr_ 0.94, *p* = 0.013), but this association attenuated after adding baseline physical function into the model. Sedentary behavior, standing, and time in bed were not associated with changes in QoL. Theoretical reallocation of 30 min of physical activity into sedentary behavior, standing or time in bed was estimated to decrease QoL by 0.5 (CI 95% -0.6 to -0.4), 0.6 (-0.7 to -0.5) and 0.4 (-0.5 to -0.3) points, respectively.

**Conclusions:**

Theoretical reallocation of physical activity into sedentary behavior, standing, and time in bed was found to be associated with prospective decline in QoL among older adults. Engaging more in physical activity and less in more passive activities may promote better QoL with advancing age.

**Supplementary Information:**

The online version contains supplementary material available at 10.1186/s12966-024-01681-9.

## Background

Quality of life (QoL) is a multidimensional and broad concept that reflects elements of life that hold significance to an individual. World Health Organization defines quality of life as an individual’s perception of their position in life in the context of the culture and value systems in which they live, and in relation to their goals, expectations, standards, and concerns [1]. It encompasses various domains such as physical and mental well-being, and interpersonal connections. Alas, aging is often accompanied with the increase of multimorbidity and loss of physical functioning, that may negatively impact QoL [2].

Movement behaviors, comprising physical activity, sedentary behavior, and sleep, can affect the maintenance of QoL with advancing age. Especially higher moderate-to-vigorous physical activity has been consistently linked with better QoL in previous studies [3, 4]. Better sleep quality has also been associated with better QoL [5], but findings on whether sleep duration is also associated with QoL are somewhat inconsistent. Particularly extreme sleep durations (short or long) may be associated with poorer QoL [3, 6, 7]. Furthermore, prolonged sedentary time may also decrease QoL with advancing age [3, 8–10], but findings are inconclusive with other authors not reporting an association between sedentary behavior and QoL [3, 6, 11].

Most of these earlier studies focusing on movement behaviors and QoL in old age, however, study physical activity, sedentary behavior, and sleep as individual behaviors rather than codependent behaviors that all happen within the constraints of the 24-hour day. In time-use epidemiology, the focus has shifted from individual behaviors into finding the optimal balance between all movement behaviors [12, 13]. Increasing time-use in one movement behavior (e.g., physical activity) will necessarily decrease time-use in at least one other movement behavior (e.g., sedentary behavior), and thus, many of the health benefits of physical activity are likely explained by simultaneous changes in several movement behaviors. Although there is a growing body of literature targeting the association of daily composition of movement behaviors with health outcomes [14–19], to our knowledge only one previous cross-sectional study explored the association between QoL and the entire time-use composition in movement behaviors in old age [20]. Therefore, the aim of the present study is to explore whether 24-hour movement behavior composition can predict longitudinal changes in QoL among community-dwelling older adults over a four-year follow-up period.

## Methods

The present study is part of the Active aging - resilience and external support as modifiers of the disablement outcome (AGNES) project and involves individuals aged 75, 80, and 85 residing in the Jyväskylä region of Central Finland. The initial sample was drawn from the Digital and Population Data Services Agency. In 2017–2018, all people born in 1942, 1938, and 1933 were invited to participate. In 2018, an additional sampling was done and people born in 1943, 1939, and 1934 were also invited to participate. Among the younger cohorts, we randomly selected approximately half of them, while all of those in the oldest cohort were invited. In total, 2791 people were invited to participate [22]. Inclusion criteria were willingness to participate and ability to communicate [21]. From 2017 to 2018, a total of 1021 individuals underwent a home interview, of whom 495 agreed to wear two tri-axial accelerometers for 7–10 consecutive days following the interview, of whom 433 had at least 3 days of data on both accelerometers [22]. Four-year follow-up data collection was conducted from 2021 to 2022. Of the 433 participants who wore both accelerometers at baseline, 68 did not want to participate the follow-up measurements, 16 were deceased, and 29 were excluded for other reasons (not reached, missing data etc.). Participants with valid accelerometer data from both devices for a minimum of 3 days, accurate detection of bed- and wake times for at least 3 nights, and complete data on QoL in both time points formed the analytical sample of the present study (*n* = 203).

### 24-hour movement behaviors

One accelerometer was attached to the participants’ dominant thigh (UKK RM42; UKK Terveyspalvelut Oy, Tampere, Finland, ± 16 g range, 13-bit analog-to-digital conversion, sampled at 100 Hz), while the other was placed on the chest (either sternum or the left side of the chest if sternum placement was uncomfortable due to e.g., soft tissues. eMotion Faros 180, Bittium Corporation, Oulu, Finland, ± 16 g range, 14-bit, sampled at 100 Hz). Accelerometer data were processed in non-overlapping 5-second epochs. Angle of the device with respect to the orientation during walking was estimated (0 = aligned with the orientation during walking, π/2 = 90 degree tilt, π = upside down), and used for posture estimation as follows: lying if both accelerometers indicated an angle > π/4, sitting if the thigh-worn accelerometer indicated an angle > π/4 and the trunk-worn accelerometer indicated an angle of ≤ π/4, and upright if both accelerometers indicated an angle of ≤ π/4 [23]. From the data collected by the accelerometers, the mean amplitude deviation (MAD = 1/n *∑ |rk –r|) of each 24-hour period was calculated based on the vector magnitude (Euclidian norm) of the resultant acceleration (√x2 + y2 + z2) in non-overlapping 5-second epochs, following the methodology outlined in previous studies [24].

Bedtimes and arising times were estimated using accelerometer recordings using an automated algorithm by van der Berg and colleagues [25] and they were used to calculate time in bed (TIB). The algorithm has been described in detail elsewhere [25]. Briefly, bedtimes were determined by summing up consecutive bouts of lying down and comparing them against pre-set time-based cut-off values which depended on the start of the first bout. When the sum exceeded the pre-determined cut-off value, bedtime was determined at the start of the first bout. Similarly, arising times were determined based on summing up active bouts and comparing them against pre-set cut-off values. The original algorithm was slightly modified, so that TIB was restricted to lying down rather than allowing either sitting or lying down postures. All bedtimes and arising times that we derived utilizing the modified algorithm were visually confirmed. Bed- and arising times, that were deemed as invalid based on visual inspection (13% of bedtimes and 9% of arising times), were excluded from further analysis. Waking hours were also determined based on bedtimes and arising times, and then categorized into physical activity, standing and sedentary behavior based on posture and signal intensity. Physical activity was determined as epochs with a MAD threshold of ≥ 0.035 units of gravity (g) and upright posture, standing as epochs < 0.035 g and upright posture, and sedentary behavior as epochs in sitting or lying posture [23]. The threshold of 0.035 g was determined through laboratory experimentation [26].

### Quality of life

Quality of life was assessed at baseline and follow-up with the short version of the Older People’s Quality of Life Questionnaire (OPQOL-brief) which has been found to be a reliable and valid measure of QoL among older adults [27]. The questionnaire includes 13 items on perceived satisfaction with prospects, health, social relationships, financial situation, activity, home, safety, leisure, and life overall. All questions are assessed with the scale from 1 to 5 (strongly disagree to strongly agree). Individual scores were summed up into a sum score ranging from 13 to 65 (higher scores indicate better QoL).

### Covariates

Participants’ sex and age were drawn from the Finnish Digital and Population Data Services Agency. Education was assessed as self-reported years of education. Morbidity was measured as the number of chronic diseases diagnosed by a physician and assessed using a self-reported questionnaire [21]. Depressive symptoms was measured with the Center for Epidemiologic Studies Depression Scale (CES-D) [28]. The range for CES-D is 0–60 and higher scores indicate more depressive symptoms. Physical function was assessed with the Short Physical Performance Battery (SPPB) which includes tests of balance, walking speed and chair stands [29]. The range for SPPB is 0–12 and higher scores indicate better physical performance.

### Statistical analysis

#### Descriptive statistics

Participant characteristics were described as means with standard deviations for continuous variables and percentages for categorical variables. Differences in the descriptive characteristics of participants according to tertiles of QoL at follow-up were studied with Chi-Square test for categorical variables and Kruskal-Wallis test for continuous variables. To address the effect of loss to follow-up and missing data on our findings, we compared the descriptive characteristics of our analytical sample with excluded participants from the original sample wearing both accelerometers at baseline (*n* = 433).

#### Compositional analyses

Compositional geometric means of the movement behaviors were calculated to describe the average composition of 24-hour movement behaviors. For the compositional multivariate linear regression analyses, time spent in PA, standing, SED and TIB were transformed into three isometric log-ratio (ilr) coordinates according to the following equations [14, 30]:

$$\:{z}_{1}=\:\sqrt{\frac{3}{4}}\text{ln}\frac{PA}{\sqrt[3]{\:STA\:x\:SED\:x\:TIB\:}}\:\:\:\:$$

$$\:{z}_{2}=\:\sqrt{\frac{2}{3}}\text{ln}\frac{STA}{\sqrt[2]{SED\:x\:TIB\:}}\:\:\:\:\:$$

$$\:{z}_{3}=\:\sqrt{\frac{1}{2}}\text{l}\text{n}\frac{SED}{\sqrt[1]{\:TIB}}$$

The first ilr-coordinate z_1_ represents PA in relation to the compositional average of the remaining movement behaviours. The second ilr-coordinate z_2_ represents the balance between standing and the compositional average of SED and TIB. The final ilr-coordinate z_3_ represents the balance between SED and the compositional average of TIB. In total, four sets of ilr-coordinates were formed in a way that each component was put in the first position of the composition.

The daily composition expressed as ilr-coordinates were then used as predictors of QoL at baseline and follow-up in the compositional linear regression models. Model 1 was adjusted for age and sex, model 2 for age, sex, education, chronic conditions, and depressive symptoms, and model 3 for age, sex, education, chronic conditions, depressive symptoms, and physical function. All prospective models were additionally adjusted for baseline QoL. The linear regression model was repeated for all sets of ilr-coordinates to study the relative importance of each part of the composition in relation to the remaining movement behaviors. The associations were presented as beta coefficients and their 95% confidence intervals. The beta coefficients indicate the change in QoL for each one-unit ilr increase, thus pointing out to presence of association but effect sizes cannot be drawn directly from the beta coefficients.

To illustrate the range of optimal time-use composition for the maintenance of QoL over time, a heat map was plotted for different combinations of time in physical activity, sedentary behavior and standing when TIB was fixed to 8 h following an approach by Chastin and colleagues and McGregor and colleagues [12, 31]. Again, different combinations of movement behaviors were transformed into ilr-coordinates and QoL was estimated based on the regression model adjusted for age, sex, and baseline QoL score. The range of values were based on the distribution of physical activity, sedentary behavior and standing among the study group.

Finally, to aid interpretation of the findings, isotemporal substitution models were used to estimate the effect of one-to-one time reallocations between daily movement behaviors on QOL over the follow-up [32]. Reallocations between movement behaviors were calculated based on the mean composition at baseline (100 min of physical activity, 228 min of standing, 536 min of TIB, 556 min of sedentary behavior), and calculated as reallocations of 10, 30 and 60 min between each movement behavior. The hypothetical reallocated compositions were transformed into ilr-coordinates and changes in QoL after each reallocation were estimated based on the fully adjusted regression model (model 3). The results are shown as estimated changes in QoL and their 95% confidence intervals (CI). Statistical analyses were performed using the SPSS statistical software package (IBM SPSS Statistics Version 28.0, IBM Corp, Armonk, NY) and the “R” statistical environment (version 4.3.1).

## Results

The participants mean age at baseline was 77.5 (SD 2.8) years and 62% were women. During the 4-year follow-up, QoL scores decreased by 5% (Mean − 2.8, SD 4.5 points) on average. The mean follow-up time for participants was 3.9 (SD 0.3) years. Descriptive characteristics of participants are presented in Table 1 according to QoL tertiles at follow-up. Participants in the low QoL group at follow-up were more likely women (*p* = 0.034), had lower education (*p* = 0.019), more chronic diseases (*p* = 0.012), more depressive symptoms (*p* < 0.001), and lower physical function (*p* < 0.001) at baseline than those with higher QoL. The mean composition of participants was 100 min of physical activity, 228 min of standing, 536 min of TIB and 556 min of sedentary behavior per day. Compared to the excluded participants of the full sample wearing both accelerometers at baseline (*n* = 433), the participants included in the present analyses were younger (*p* < 0.001), more years of education (*p* = 0.046), fewer depressive symptoms (*p* = 0.001), better physical function (*p* = 0.024), and better QoL at baseline (*p* = 0.023, Supplementary Table 1).

**Table 1:** Descriptive characteristics of participants according to QoL tertiles at follow-up

	All (*n* = 203)	Low tertile (*n* = 70)	Middle tertile (*n* = 65)	High tertile (*n* = 68)	*p*-value
Women, % (*n*)	62 (125)	71 (50)	63 (41)	50 (34)	**0.034**
Age at BL, years, mean (SD)	77.5 (2.8)	78.0 (2.8)	77.5 (3.0)	76.9 (2.4)	0.167
Education, years, mean (SD)	12.2 (4.5)	11.2 (4.2)	11.9 (4.2)	13.4 (4.8)	**0.019**
Chronic diseases, *n*, mean (SD)	3.1 (1.9)	3.6 (1.9)	2.8 (1.8)	2.8 (1.9)	**0.012**
CES-D, score, mean (SD)	6.5 (5.6)	8.8 (6.3)	6.2 (5.4)	4.4 (4.0)	**< 0.001**
SPPB, score, mean (SD)	10.6 (1.6)	9.8 (2.0)	10.9 (1 L.3)	11.2 (1.1)	**< 0.001**
QoL at BL, mean (SD)	56.2 (4.9)	52.9 (4.4)	56.4 (4.2)	59.3 (3.8)	**< 0.001**

Note; BL = baseline, CES-D = Center for Epidemiologic Studies Depression Scale, SPPB = Short Physical Performance Battery; Low QoL tertile < = 51, Middle tertile = 52–55, High tertile > = 56

The results of compositional linear regression analyses are presented in Table 2. In the cross-sectional analyses, higher proportion of physical activity (β_ilr_ 1.30, *p* = 0.001) and TIB (β_ilr_ 2.72, *p* = 0.005) relative to the remaining movement behaviors were associated with better QoL. The associations remained after adjusting for education, depressive symptoms, and chronic conditions. Conversely, higher standing (β_ilr_ -1.99, *p* < 0.001) and sedentary behavior (β_ilr_ -2.13, *p* = 0.004) relative to remaining behaviors were associated with lower QoL. In the final model including physical function, all associations attenuated. In the longitudinal analyses, higher baseline physical activity relative to remaining behaviors was associated with better maintenance of QoL at follow-up (β_ilr_ 0.94, *p* = 0.013) and the association remained after adjusting for education, depressive symptoms, and chronic conditions. However, after adding physical function into the model the association attenuated (β_ilr_ 0.66, *p* = 0.094). Standing, sedentary behavior and TIB were not associated with changes in QoL in the longitudinal analyses.

**Table 2:** Compositional linear regression analyses on cross-sectional and longitudinal associations between baseline time-use composition and QoL

Cross-sectional	Model 1			Model 2			Model 3		
	βilr	95% CI	*p*-value	βilr	95% CI	*p*-value	βilr	95% CI	*p*-value
PA vs. other behaviors	1.30	0.52 to 2.09	**0.001**	0.83	0.10 to 1.56	**0.025**	0.03	-0.66 to 0.73	0.924
Standing vs. other behaviors	-1.99	-2.88 to -0.90	**< 0.001**	-1.39	-2.28 to -0.49	**0.003**	-0.70	-1.47 to 0.07	0.074
Sedentary vs. other behaviors	-2.13	-3.57 to -0.70	**0.004**	-1.63	-2.92 to -0.33	**0.014**	-0.41	-1.58 to 0.76	0.494
TIB vs. other behaviors	2.72	0.82 to 4.62	**0.005**	2.18	0.47 to 3.89	**0.013**	1.08	-0.42 to 2.58	0.158
**Longitudinal**									
PA vs. other behaviors	0.94	0.20 to 1.67	**0.013**	0.81	0.05 to 1.57	**0.036**	0.66	-0.11 to 1.42	0.094
Standing vs. other behaviors	-0.79	-1.73 to 0.15	0.098	-0.71	-1.65 to 0.22	0.137	-0.68	-1.62 to 0.25	0.151
Sedentary vs. other behaviors	-0.70	-2.05 to 0.64	0.305	-0.62	-1.97 to 0.73	0.369	-0.42	-1.77 to 0.94	0.543
TIB vs. other behaviors	0.56	-1.22 to 2.34	0.536	0.52	-1.26 to 2.30	0.565	0.45	-1.32 to 2.22	0.619

Note; Results shown only for the first ilr-coordinate, i.e. the identified behaviour versus all the others. Model 1 adjusted for age and sex, Model 2 adjusted additionally for education, baseline chronic conditions and depressive symptoms, All prospective analyses additionally adjusted for baseline quality of life, Model 3 additionally adjusted for baseline physical function, PA = physical activity, TIB = time in bed

**Fig. 1:**
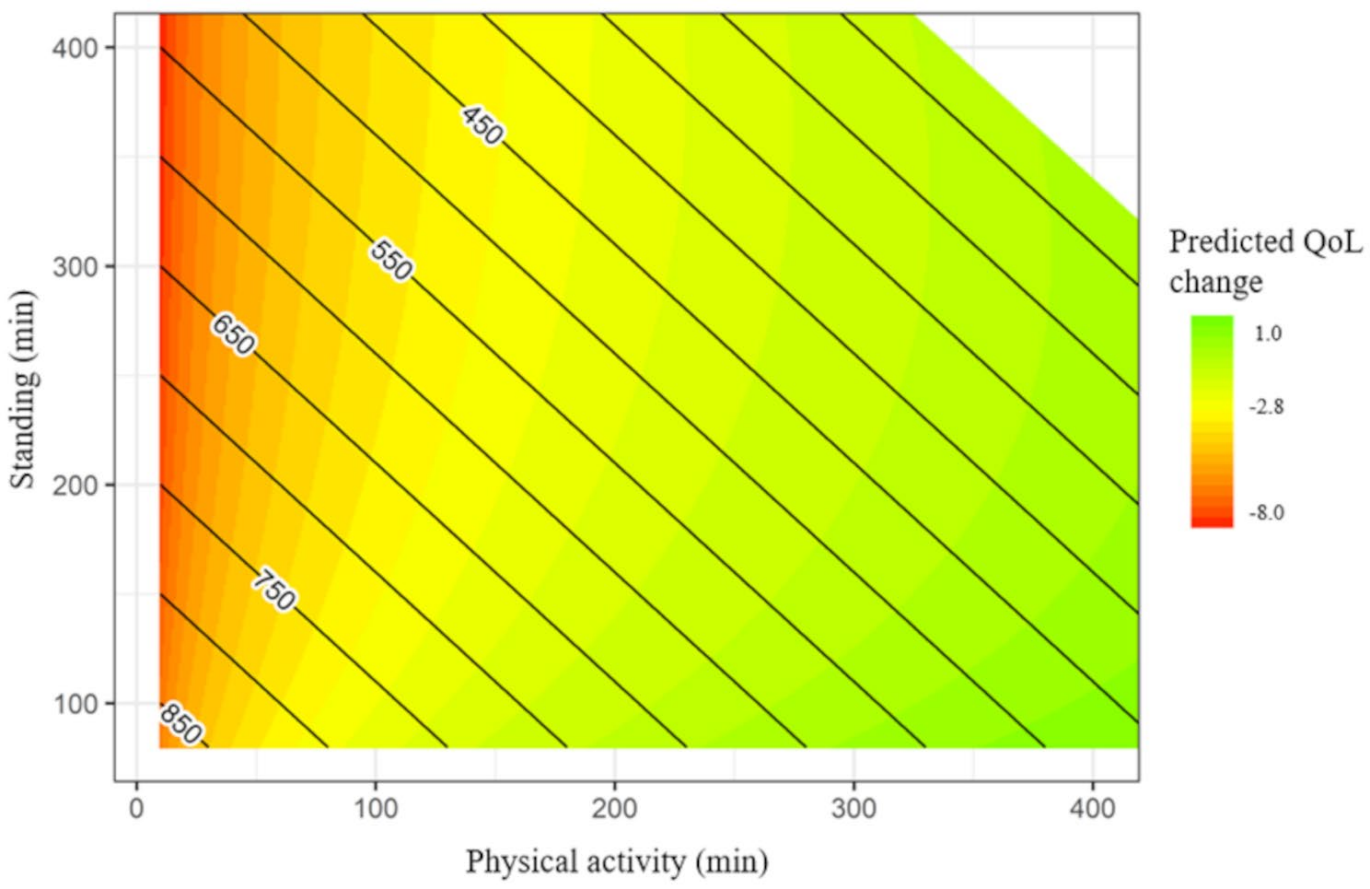
Heatmap showing estimated QoL change at follow-up with different balances between PA, STA, and SED Note; x-axis: physical activity (PA) minutes, y-axis: standing (STA) minutes, contour lines: sedentary behavior (SED) minutes. Red color reflects greater than average estimated decrease in QoL (-2.8 units), yellow color average estimated decrease, and green color lower than average estimated decrease or increase in QoL

Figure 1 illustrates estimated QoL at follow-up with a range of different combinations of physical activity, sedentary behavior, and standing. The risk for poorer QoL was the highest with combinations including low physical activity and high standing, or sedentary behavior, but changes in QoL seem to be especially driven by changes in physical activity minutes. A lower-than-average estimated decrease in QoL scores was achieved with different daily combinations including high daily physical activity minutes and low-to-moderate standing and sedentary behavior.

**Fig. 2:**
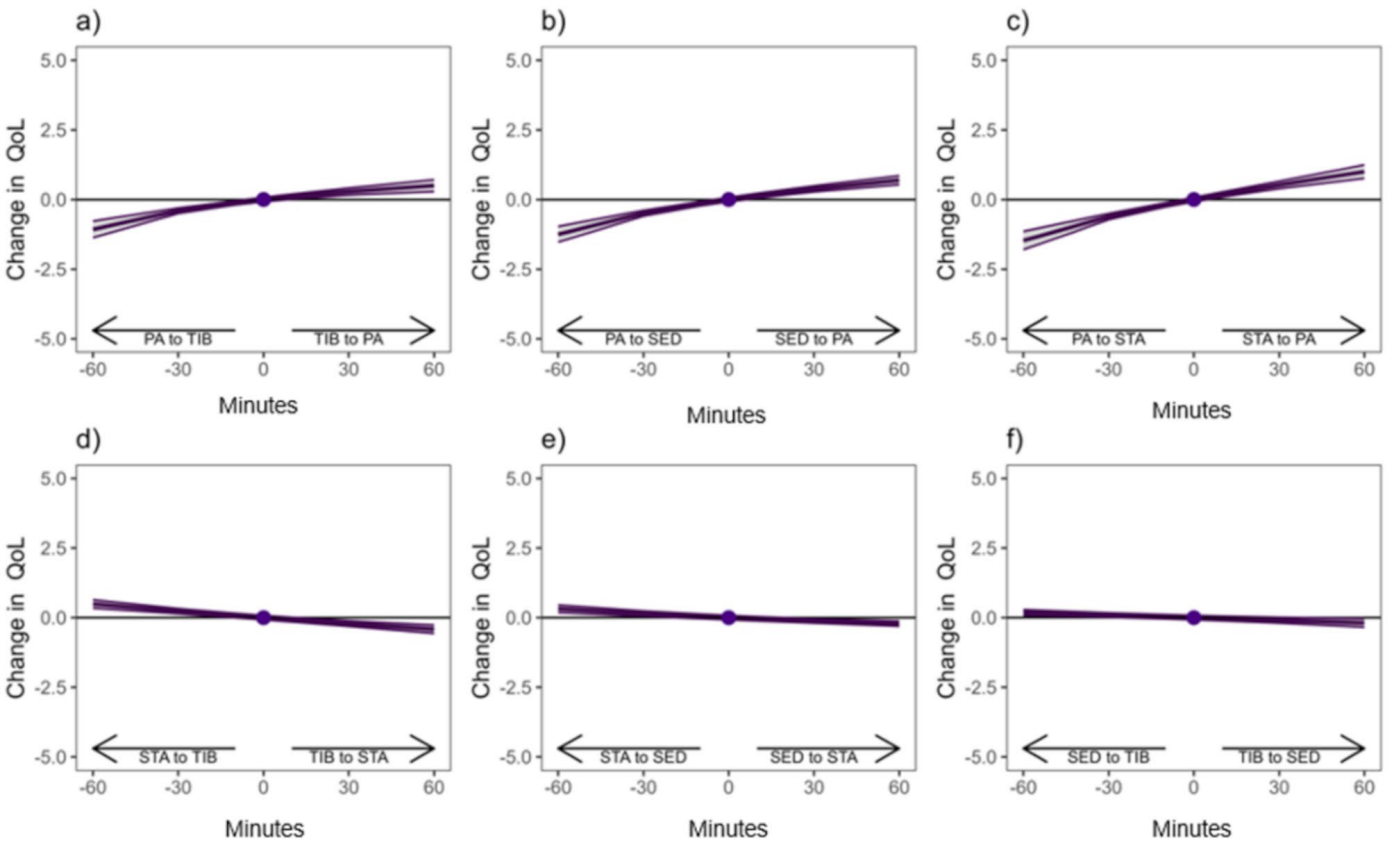
One-to-one reallocations of physical activity, standing, sedentary behavior, and time in bed Note; PA = physical activity, STA = standing, SED = sedentary behavior, TIB = time in bed. The dot represents the mean baseline composition of 100 min of PA, 228 min of STA, 556 min of SB, 536 min of TIB and QoL score of 52.6

Finally, the effects of theoretical one-to-one time reallocations between each movement behavior on QoL at follow-up are illustrated in Fig. 2. Theoretical reallocation of time from physical activity into any other behavior was associated with an estimated decrease in QoL (Fig. 2a-c). Similarly, reallocating time into physical activity from any other behavior was associated with an estimated increase in QoL, but the changes in QoL were slightly smaller in absolute terms when compared to reallocation of time away from physical activity. Furthermore, reallocating time from standing into TIB and sedentary behavior was associated with a small estimated increase in QoL at follow-up (Fig. 1d & f).

## Discussion

The main finding of the study was that theoretical time reallocation from physical activity into any other movement behavior predicted decrease in QoL over time among community-dwelling older adults, while reallocating time into physical activity was associated with better maintenance of QoL. Theoretical reallocation of time from physical activity into stationary waking behaviors, namely sedentary behavior and standing, was associated with slightly greater decreases in QoL than reallocating time into TIB. Time reallocation from physical activity into other movement behaviors was associated with slightly greater change in QoL than reallocating time from other behaviors into physical activity. Furthermore, the association of physical activity relative to all remaining movement behaviors in the linear regression models attenuated after adding physical function into the model suggesting it may explain the observed associations. Although the estimated changes on QoL were rather modest, we postulate that preventing decline in physical activity with advancing age may support the maintenance of better QoL over time. Since QoL reflects elements of life that individuals’ hold significance to, even small changes in it can be considered important. The findings of this study extend previous cross-sectional findings on the association between 24-hour movement behaviors and QoL by replicating the findings in a prospective investigation.

Our findings suggest that the proportion of physical activity rather than sedentary behavior, standing or time in bed predicts changes in QoL. This was also seen in the heat map which illustrated that different combinations of daily waking time-use including high level of physical activity was associated with more favorable changes in QoL. The finding is consistent with previous cross-sectional findings showing that a theoretical reallocation of 30 min into physical activity was associated with better QoL in old age [20]. The cross-sectional association between physical activity and QoL was explained mainly by baseline physical function, and the longitudinal association of physical activity relative to all other behaviors also attenuated after adding physical function into the model. However, the longitudinal one-to-one theoretical time reallocations the association remained in the fully adjusted regression model. These observations suggest that the longitudinal associations may also be explained by maintenance of physical function and health. While better physical function enables engagement in higher levels of physical activity, physical activity is also an important in the maintenance of better physical function with advancing age [33]. Furthermore, we found that decreasing time-use in physical activity with respect to any other movement behavior was associated with a higher estimated change in QoL than increasing time-use in physical activity, which is parallel to earlier findings [20]. This observation is likely explained in part by the relatively high proportion of physical activity in the mean composition (100 min), which may partly be due to the short epoch length in the accelerometry analysis. Older individuals that already engage in relatively high level of physical activity may be less likely to receive additional benefits of further increasing daily time-use in physical activity on their QoL than older adults who accumulate low physical activity levels. This, however, need to be confirmed in future studies including more inactive older adults.

In the present analyses, standing seemed to be the movement behavior with the most detrimental association with QoL. Notably, standing is often included as light physical activity due to the upright posture and increased energy consumption [34, 35]. QoL is a broad concept, and different factors contribute to QoL between individuals based on factors which they hold value to. Activities that take place while standing may often include household chores that may be seen as obligatory routines rather than enjoyable activities, thus potentially decreasing QoL. Moreover, although apparently light, standing may decrease the likelihood of engaging in other more intensive activities later on in the day. Replacing sedentary behavior and TIB with standing was associated with an estimated decrease in QoL in the isotemporal substitution models, but this change was minimal and unlikely to have any real-life significance. Whether or not standing displaces more intensive movement behavior remains to be explored in future studies.

The main strengths of the study include the longitudinal study design and continuous monitoring of physical activity, sedentary behavior and TIB for 3–7 days, which allowed us to account for day-to-day variations in 24-hour movement behaviors. Another strength was the availability of two concurrently worn tri-axial accelerometers. This allowed us to gain valid information of a larger variety of body postures and separate lying down from sitting, which would not be reliable using a single wearable sensor. There are also limitations that should be considered when interpreting the findings. First, we only accounted for the 24-hour movement behaviors at baseline, and thus, time reallocations were based on estimated changes, and not actual changes, in the daily time-use composition. Furthermore, although physical activity has been consistently associated with improved quality of life [3], we cannot be certain whether higher QoL may also lead to higher physical activity levels. Second, although we used a validated algorithm to identify bed- and wake times, we were not able to separate actual sleep state from wake. Thus, our estimate of TIB can also include periods of wake time while lying in bed. Furthermore, we did not assess sleep quality, which may be more important with regards to QoL than sleep duration [5]. Third, it should be noted that the COVID-19 pandemic occurred during the follow-up period of the study. During the first wave and social distancing physical activity temporarily increased [36] while QoL slightly decreased [37], which may have impacted our findings. Finally, the older adults included in the present analyses had better physical functioning and were physically more active compared with the entire AGNES sample [22] which limits the generalizability of the present findings.

## Conclusions

In conclusion, the findings indicate that reallocating time from physical activity into other movement behaviors is associated with a decrease in QoL over time among older adults. In contrast, engaging more in physical activity and less in stationary activities may promote better QoL with advancing age. Especially preventing reduction in physical activity and simultaneously decreasing time-use in more passive waking behaviors could be encouraged for older adults and might warrant further exploration in a randomized controlled trial.

## Electronic supplementary material

Below is the link to the electronic supplementary material.

Supplementary Material 1

Supplementary Material 2

## Data Availability

After completion of the study, data will be stored at the Finnish Social Science Data Archive without potential identifiers (open access). Until then, pseudonymized datasets are available to external collaborators subject to agreement on the terms of data use and publication of results. To request the data, please contact Professor Taina Rantanen (taina.rantanen@jyu.fi).

## Abbreviations

AGNES: Active agingresilience and external support as modifiers of the disablement outcome
CES-D: Center for Epidemiologic Studies Depression Scale
CI: Confidence interval
MAD: Mean Amplitude Deviation
PA: Physical activity
SED: Sedentary behavior
SPPB: Short Physical Performance Battery
STA: Standing
TIB: Time in bed
QoL: Quality of Life
