# Supplementary material for: 24-hour movement behaviors and changes in quality of life over time among community-dwelling older adults: a compositional data analysis
Source: Int J Behav Nutr Phys Act. 2024 Nov 12;21:130. doi: 10.1186/s12966-024-01681-9 (PMC11555968; doi:10.1186/s12966-024-01681-9)
Supplement: Supplementary file 1 — Supplementary Material 1 [file 12966_2024_1681_MOESM1_ESM.docx]

Supplementary table 1**.** Comparison of baseline characteristics between the full sample and study participants

|  | Full sample  (n=433) | Excluded  (n=230) | Included  (n=203) | p-valuea |
| --- | --- | --- | --- | --- |
| Women | 60 % | 58 % | 62 % | 483 ^a^ |
| Age at BL, years, mean (SD) | 78.3 (3.4) | 79.0 (3.8) | 77.5 (2.8) | **<0.001 ^b^** |
| Education, years, mean (SD) | 11.7 (4.2) | 11.4 (4.0) | 12.2 (4.5) | **0.046 ^b^** |
| Chronic diseases, n, mean (SD) | 3.2 (2.0) | 3.3 (2.1) | 3.1 (1.9) | 0.388 ^b^ |
| CES-D, score, mean (SD) | 7.7 (6.6) | 8.7 (7.3) | 6.5 (5.6) | **0.001 ^b^** |
| SPPB, score, mean (SD) | 10.3 (1.9) | 10.1 (2.1) | 10.6 (1.6) | **0.024 ^b^** |
| QoL at BL, mean (SD) | 55.5 (5.4) | 54.8 (5.8) | 56.2 (4.9) | **0.023 ^b^** |

Note. ^a^ Pearson Chi-Square ^b^ Independent-Samples Mann-Whitney U (Wilcoxon Rank-Sum) Test
